# Histopathologic Feature of Hyalinization Predicts Recurrence of Conventional/Solid Multicystic Ameloblastomas

**DOI:** 10.3390/diagnostics12051114

**Published:** 2022-04-29

**Authors:** Dominic Augustine, Roopa S. Rao, Lakshminarayana Surendra, Shankargouda Patil, Thuckanaickenpalayam Ragunathan Yoithapprabhunath, Sarah Albogami, Shaheen Shamsuddin, Sulphi Abdul Basheer, Shan Sainudeen

**Affiliations:** 1 Department of Oral Pathology & Microbiology, Faculty of Dental Sciences, Ramaiah University of Applied Sciences, MSR Nagar, Bengaluru 560054, Karnataka, India; dominic2germain@gmail.com (D.A.); drroopasrao1971@gmail.com (R.S.R.); drsuri29@gmail.com (L.S.); 2 Department of Maxillofacial Surgery and Diagnostic Sciences, Division of Oral Pathology, College of Dentistry, Jazan University, Shwajra Campus, P.O. Box 114, Jazan 45412, Saudi Arabia; 3 Department of Oral and Maxillofacial Pathology, Vivekanandha Dental College for Women, Elaiyampalayam 637205, Tamil Nadu, India; yoitha.dentist@gmail.com; 4 Department of Biotechnology, College of Science, Taif University, P.O. Box 11099, Taif 21944, Saudi Arabia; dr.sarah@tu.edu.sa; 5 Department of Orthodontics, College of Dentistry, King Khalid University, P.O. Box 3263, Abha 61471, Saudi Arabia; drshaheenvs@hotmail.com; 6 Department of Oral and Maxillofacial Surgery, College of Dentistry, King Khalid University, P.O. Box 3263, Abha 61471, Saudi Arabia; sulphi@gmail.com; 7 Department of Restorative Dentistry, College of Dentistry, King Khalid University, P.O. Box 3263, Abha 61471, Saudi Arabia; shan@kku.edu.sa

**Keywords:** hyalinization, solid multicystic ameloblastoma, follicular ameloblastoma, plexiform ameloblastoma, recurrence, curettage, segmental resection

## Abstract

The histologic properties of tumors seem to affect their biological behavior, and the same holds good for solid multicystic ameloblastoma (SMA), a benign, locally destructive lesion. Hyalinization is one such histological factor that has been demonstrated to correlate with the biological behavior of neoplasms. The present study aimed to analyze the correlation between the severity of hyalinization (SOH) and the recurrence potential of SMAs. The study was performed on formalin-fixed, paraffin-embedded (FFPE) diagnosed archival cases of SMA, follicular SMA (*n* = 35) and plexiform SMA (*n* = 25). The cases were evaluated for SOH and scored from 0–3, and the correlation between SOH and recurrence was analyzed for statistical significance. The clinical parameters of the lesion were analyzed for statistical correlation with recurrence. The SOH significantly correlated with the recurrence of SMA (*p* = 0.001). The histologic type did not influence the biological behavior of SMA. The location of SMA in the body of the mandible (*p* = 0.036), multilocular radiolucency (*p* = 0.001) and root resorption (*p* = 0.002) also showed strong statistical correlation with recurrence. It is evident from the present study that hyalinization strongly correlates with the biological behavior of SMA. Future studies with advanced investigations could validate the presence of hyalinization and identify the origin of the hyalinized product in SMAs.

## 1. Introduction

Hyalinization is recognized as a condition in which normal tissue deteriorates into a homogeneous, translucent material [1]. The phenomenon of hyalinization has remained a subject of much debate. The origin of hyaline material has also been the subject of speculation in different pathological lesions. Odontogenic tumors are a group of heterogeneous diseases that range from hamartomatous/developmental to benign or malignant tumors with destructive potential. They make up for approximately 3% of all head and neck biopsies received [2,3].

Considerable debate has existed correlating the histologic type of SMA to its aggressive potential. Hence, other histologic parameters such as hyalinization can be assessed to resolve this dilemma.

Varied biological behavior in SMA is challenging to predict, as lesions can appear as well-defined radiolucencies less than 2 cm in diameter or as large lesions crossing the midline, causing significant facial asymmetry. Owing to its high recurrence potential, wide resective surgery is the mainstay of therapy for SMA. Wide resective surgery is associated with a low recurrence rate of 13–15%. A high rate of recurrence (90–100%) in SMA is noted when the management protocol is conservative, such as in the case of curettage [4].

Recent literature has shown that the presence of a hyalinized stroma in lesions could indicate aggressive biological behavior in oral lesions [5]. Based on this, the current study tested the hypothesis whether hyalinization in SMA could play a possible role in predicting its biological behavior. The present study aims to evaluate the presence of juxta-epithelial hyalinization in SMAs and attempts to establish a correlation between SOH and the recurrence potential of SMAs.

The results obtained could unknot potential insights on the possible role of hyalinized stroma in the aggressive biological behavior or recurrence potential of SMAs. This prior knowledge about the aggressive or recurrent potential of the lesion during the stage of incisional biopsy will warrant effective management of SMAs during their excision, ensuring better treatment outcomes for the patients. 

## 2. Materials and Methods

### 2.1. Study Design

Ethical clearance to undertake the study was obtained from the MS Ramaiah University of Applied Sciences (MSRUAS) human ethics committee, bearing number EC-2021/F/052. The study was performed on FFPE-diagnosed archival cases of SMA, follicular SMA (*n* = 35) and plexiform SMA (*n* = 25) from the department of Oral Pathology and Microbiology, Faculty of Dental Sciences, MSRUAS. Descriptive features and clinical parameters of the above cases were retrieved from archival files and tabulated. The retrieved tissues were deparaffinized by heating them at 57 °C for 10 min. Any remaining paraffin was removed using xylene. The sections were stained with Haematoxylin and Eosin. 

### 2.2. Histopathological Correlation of Hyalinization in SMA with Recurrence

The slides were randomly numbered and examined by two pathologists (DA and SL) to evaluate the SOH. In cases of ambiguity, a third pathologist (RSR) assessed the histopathological feature of hyalinization and its severity. The histologic type of SMA was also noted and tabulated. The correlation between SOH and the recurrence in SMA was statistically analyzed for significance. A subgroup comparison (follicular SMA vs. plexiform SMA) was also performed.

### 2.3. Clinicopathologic Correlation of SMA with Recurrence

The clinical parameters of size (1–2 cm = 1, 3–4 cm = 2, >4 cm = 3), jaw involved (maxilla/mandible), site, radiographic features (multilocular/unilocular), cortical expansion, and root resorption were analyzed for statistical correlation with recurrence. 

### 2.4. Interpretation and Analysis

The SOH was recorded in the H and E stained slides of SMA and scored as 0 = absent, 1 = mild, 2 = moderate and 3 = intense. Photomicrographs of the slides were captured at ×100 magnification using an Olympus Optical Microscope BX53F2, Tokyo, Japan. A chi-square test was used to compare the level of SOH and samples of SMA with and without recurrence. Logistics regression analysis and an odds ratio was determined to analyze a predictor (severity of hyalinization) of recurrence. 

## 3. Results

### 3.1. Correlation of Histological Parameter with SOH

**Figure 1:**
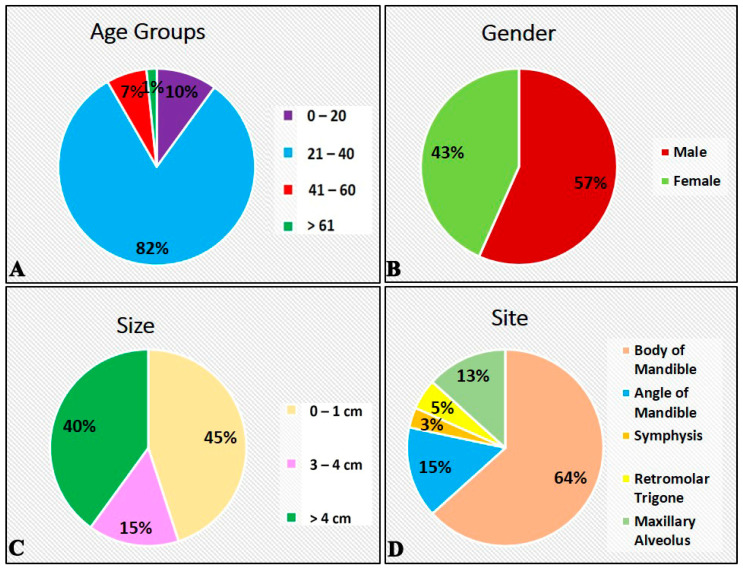
Descriptive features of participants with solid multicystic ameloblastoma. (**A**) Age distribution. (**B**) Gender distribution. (**C**) Size distribution. (**D**) Site distribution.

The descriptive features of SMA (*n* = 60) are shown in Figure 1 and Table 1. 

**Figure 2:**
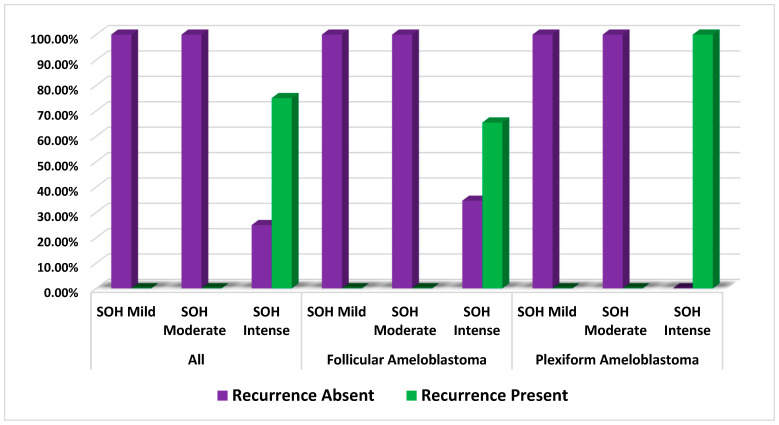
Comparison of correlation of SOH with recurrence of solid multicystic ameloblastoma.

When the histopathologic feature of sub-epithelial hyalinization was correlated with recurrence, it was noted that SOH significantly statistically correlated with the recurrence of SMA (*p* = 0.001). When the histologic type of SMA was correlated with recurrence, it was observed that both follicular SMA (*p* = 0.003) and plexiform SMA (*p* = 0.001) showed strong statistical correlation with recurrence. (Table 2 and Figure 2) 

This indicates that the histologic subtypes whether follicular or plexiform does not influence the biological behavior of SMA. 

### 3.2. Correlation of Clinical Parameters and Recurrence

**Figure 3:**
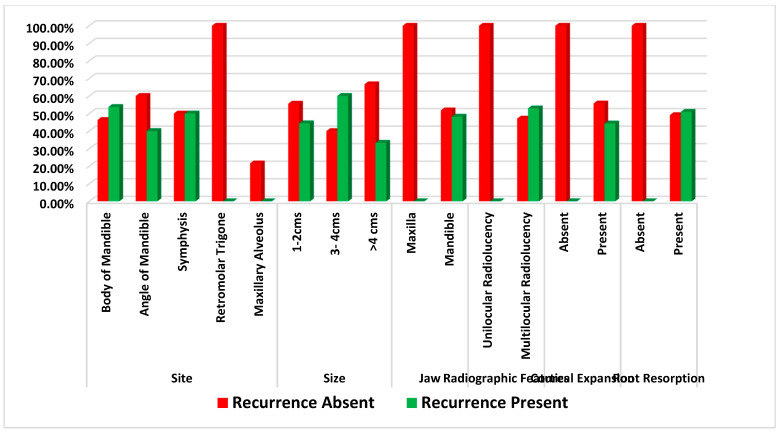
Comparison of correlation of clinical parameter with recurrence of solid multicystic ameloblastoma.

When the clinical parameters of size, jaw involved, site, cortical expansion, and root resorption were analyzed for statistical correlation with recurrence, it was observed that lesions present in the body of the mandible (*p* = 0.036), located in the mandibular jaw (*p* = 0.010), multilocular radiolucency (*p* = 0.001) and lesions with root resorption (*p* = 0.002) showed strong statistical correlation with recurrence. (Table 3 and Figure 3).

### 3.3. Logistics Regression and Odds Ratio

Table 4 shows that the odds of having recurrence is 5.0172 times greater for severe SMA, as opposed to mild SMA, and four times greater for moderate SMA compared to mild SMA.

## 4. Discussion

Hyalinization is the process of the deposition of hyaline, which is a homogenous, structureless and eosinophilic material that has been reported to be present in oral benign and malignant lesions. Concerning the maxillofacial region, reports have indicated that hyalinization in salivary gland tumors and odontogenic keratocysts strongly correlate with aggressive behavior and recurrent potential [6,7]. A single report showing the correlation of hyalinization with the recurrent potential of SMAs was published by Sathi et al. in 2008; however, the analysis was limited to only six cases [8].

The presence of hyalinization can be visualized throughout the odontogenic stroma. The connective tissue is influenced by the inductive changes which the odontogenic epithelium exerts on it. As the odontogenic epithelium is scattered throughout the stroma, we can expect hyalinization in all such areas, which makes it a diffuse phenomenon rather than a focal one. These inductive changes are characterized by hyalinization composed of connective tissue matrix proteins. The hyalinization of the stroma in the OTs indirectly indicates the hyperactivity and inductive potential of the odontogenic epithelium. Overall, we opine that hyalinization can be elicited as a diffuse change.

The above findings make hyalinization an interesting parameter to evaluate in other oral pathological lesions as well. Also, the determination of hyalinization can be done by using routine H and E stains and conventional microscopy, without the need for expensive reagents or equipment [9]. SMAs are locally aggressive, benign odontogenic tumors that can cause significant local destruction. If left untreated, they are able to reach large sizes, causing facial disfiguration and functional problems [10]. Their clinical behavior and prognosis are often unpredictable.

In 2017, the World Health Organization has re-classified ameloblastomas as conventional (solid/multicystic), peripheral and unicystic. Desmoplastic ameloblastoma was declassified as unique entity and is now considered as a histologic variant of SMA. It has also been noted that different histologic variants can be observed in the same tumor in the case of SMA [11,12,13].

Since it is clear that the histologic variant does not influence the biological behavior of SMAs, we proposed to investigate via this study whether hyalinization could be a reliable indicator of aggressive biological behavior.

**Figure 4:**
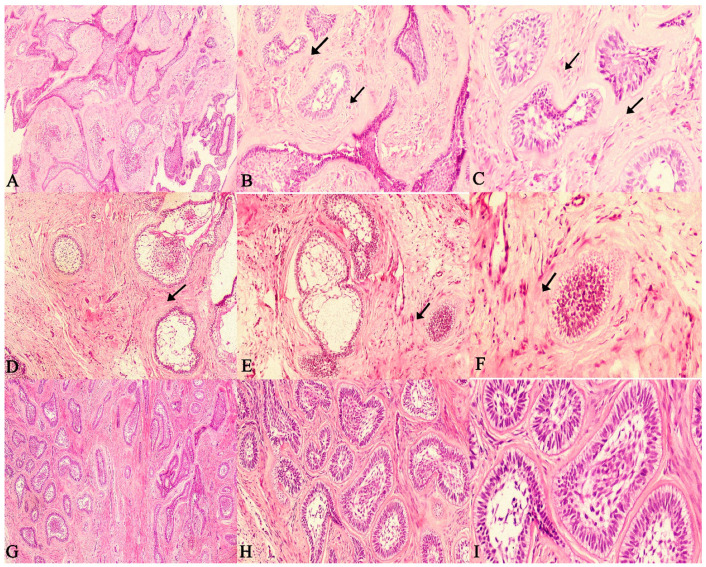
Photomicrographs of haematoxylin and eosin stain. Follicular ameloblastoma exhibiting extensive (increased SOH) juxta-epithelial and stromal hyalinization zones between follicles (**A**). 40×, (**B**). 100× and (**C**). 400× (recurrent case). Follicular ameloblastoma exhibiting (moderate SOH) juxta-epithelial and stromal hyalinization zones between follicles (**D**). 40×, (**E**). 100× and (**F**). 400× (recurrent case). Follicular ameloblastoma exhibiting (mild SOH) juxta-epithelial and stromal hyalinization zones between follicles (**G**). 40×, (**H**). 100× and (**I**). 400× (non-recurrent case). (Non-hyalinized: no evidence of hyalinization; mild: few areas of hyalinization; moderate: considerable areas of hyalinization; severe: extensive areas of Hyalinization). (Arrows indicate hyalinized areas).

**Figure 5:**
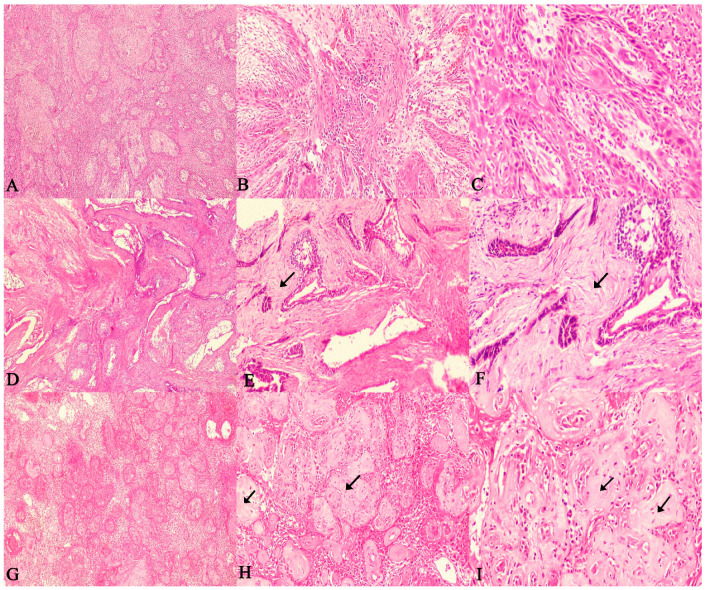
Photomicrographs of haematoxylin and eosin stain. Plexiform ameloblastoma with a non-hyalinized stroma (**A**). 40×, (**B**). 100× and (**C**). 400× (non-recurrent case). Ameloblastoma exhibiting moderate juxta-epithelial and stromal hyalinization zones between odontogenic epithelium (**D**). 40×, (**E**). 100× and (**F**). 400× (recurrent case). Plexiform ameloblastoma exhibiting extensive (increased SOH) juxta-epithelial and stromal hyalinization zones between odontogenic epithelium (**G**). 40×, (**H**). 100× and (**I**). 400× (recurrent case). (Non-hyalinized: no evidence of hyalinization; mild: few areas of hyalinization; moderate: considerable areas of hyalinization; severe: extensive areas of hyalinization). (Arrows indicate hyalinized areas).

The present study evaluated the SOH in 60 SMAs. The SOH was correlated with the recurrence of follicular and plexiform ameloblastomas and checked for statistical correlation. The comparison of correlation between SOH and the recurrence of SMA revealed a strong statistical correlation for follicular SMA (*p* = 0.003), plexiform SMA (*p* = 0.001) and with both groups combined as well (*p* = 0.001). (Figure 4A–F and Figure 5A–F). 

**Figure 6:**
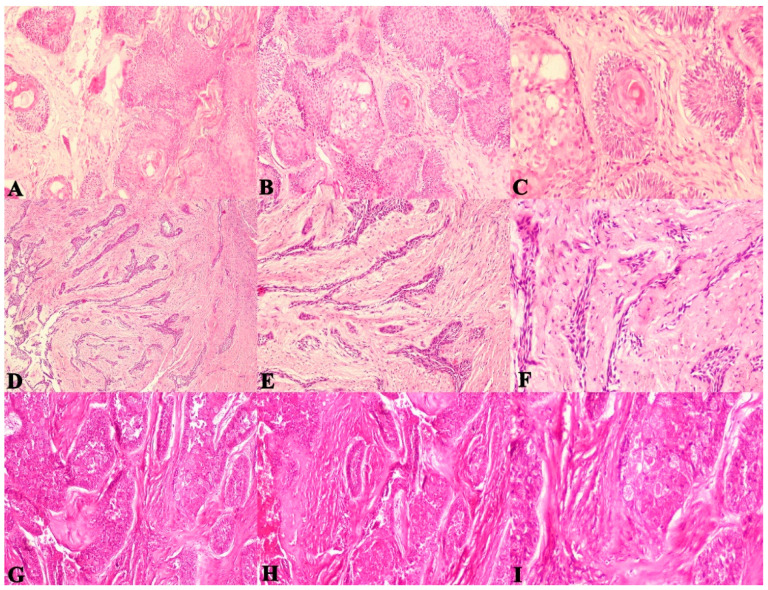
Photomicrographs of haematoxylin and eosin stain. Acanthomatous ameloblastoma exhibiting extensive hyalinization between follicles, (**A**). 40×, (**B**). 100× and (**C**). 400× Desmoplastic ameloblastoma exhibiting thin compressed strands due to desmoplastic changes and hyalinization, (**D**). 40×, (**E**). 100× and (**F**). 400× Granular cell ameloblastoma exhibiting extensive hyalinization between follicles filled with large granular cells, (**G**). 40×, (**H**). 100× and (**I**). 400×.

The majority of recurrent SMA cases showed an intense SOH. The findings also reiterate the fact that the histologic variant does not appear to influence the recurrent potential of SMAs, rather it is the feature of hyalinization that seems to distinguish aggressive and non-aggressive cases. (Figure 6A–I).

Through the Figure 4, Figure 5 and Figure 6 it is possible to demonstrate the microscopic appearance of all the histological variants of ameloblastoma that have a propensity to recur or not. Those that have a higher risk of recurrence are those that show intense stromal hyalinization. Those that are non-hyalinized have a lower recurrence rate or rather recurrence is absent. These figures make it possible to differentiate recurrent versus non recurrent SMA based on stromal hyalinization.

The presence of subepithelial and stromal hyalinization in ameloblastomas have been explained by a collection of hypotheses. It is believed that the hyalinized stroma around the odontogenic follicles is the result of tall columnar ameloblast-like cells attempting, in vain, to secrete tooth-like material as the cells are trying to complete the cycle of odontogenesis [14]. This results in the presence of an eosinophilic unmineralized matrix around the odontogenic follicles. Another explanation is that, in an effort to wall off the impending local invasion, the host responds through formation of hyalinized zones around the odontogenic follicles or tumor cells [15]. Even though routine H and E stain aided by differential stains can identify the hyalinized material, specific protein markers employed via antigen–antibody reactions such as immunohistochemistry could identify the origin of the hyalinized material. If the hyalinized material is strongly positive for collagen type 4 and laminin-5 proteins, it is indicative of secretion by the basal cells of the odontogenic follicles [16]. Conversely, if ground substance material proteins such as heparan, hyaluronic acid and chondroitin sulphate are over expressed, it can be concluded that the origin of hyaline is purely stromal [17].

A molecular assessment (Immunohistochemistry) study conducted by Sathi et al. in 2008 evaluated six cases of SMA for basement membrane molecules and apoptosis-related genes [8]. The authors reported that the hyalinized areas in all sections were strongly positive for 10E4. Tumor cells were positive for 10E4, heparinase and caspase-6. Also, CD34-positive endothelial cells within the hyalinization were absent. The authors concluded that hyalinization regulates heparan sulfate proteoglycans and curbs the functions of heparanase and prevents angiogenesis. It is postulated that hyalinization prevents stromal–tumor cell interactions, thereby inhibiting tumor growth [8].

However, the analysis was performed only on six cases, without a clinico-pathologic correlation. The current study exhibited contrasting results, as hyalinized stroma strongly correlated with aggressive biological behavior and the recurrence of SMA. It is suggested that myofibroblasts play a role in fibrosis and hyalinization. Myofibroblasts present in the stroma can facilitate tumor progression, attributed to the invasiveness of desmoplastic ameloblastoma, as proposed by Hinz, et al. in 2003 [18]. The same was suggested by Vared et al. in 2005. In their study, smooth muscle actin positive myofibroblasts were seen close to the ameloblastomatous islands. A study by Sherlin et al. in 2013 further justified the role of the myofibroblast in ameloblastoma by demonstrating smooth muscle actin positivity at the tumor front of the lesion [19].

In the present study, the significant clinical parameters were also correlated with SOH to establish a clinicopathologic correlation. It was noted that lesions present in the body of the mandible (*p* = 0.036), lesions of the mandibular jaw (*p* = 0.010), the presence of multilocular radiolucency (*p* = 0.001) and lesions with root resorption (*p* = 0.002) showed strong statistical correlation with recurrence. These results were in accordance with previously conducted studies, such as the study by Carvalho et al. (2017), who stated that most recurrent ameloblastomas are found in the posterior mandible and those with a multilocular presentation have a high preponderance for recurrence [20]. The recurrence in SMA is also correspondingly governed by the treatment modality. In 1993, an analysis of 315 cases of ameloblastoma was performed by Olaitan et al., who stated that curettage or enucleation is not recommended due to the high rate of recurrence [21]. In those patients in whom autogenous bone grafts were employed, no recurrence was observed, resulting in a 100% cure. It was demonstrated by Au et al. in 2019 that radical resection resulted in a low recurrence rate with regard to SMA [22]. From the literature review, it is clear that clinical features do not influence the recurrence of SMAs; rather, it is the management strategy alone that matters [23,24]. In the current study, logistics regression analysis and an odds ratio were determined to analyze a predictor (severity of hyalinization) of recurrence. It was observed that the odds of having recurrence are 5.0172 times greater for cases of SMA with severe hyalinization when compared to mild hyalinization.

The results of the current study show that hyalinization is a reliable indicator for the purpose of predicting the aggressive behavior or recurrence of SMA. Based on the management strategies available to treat SMA, extensively hyalinized SMAs should be considered as potentially aggressive with a high recurrent potential.

## 5. Conclusions

Hyalinization correlates strongly with the aggressive biological behavior of SMA. This finding might enable the surgeons to better evaluate the aggressive potential of SMAs and treat them accordingly. Immunohistochemical markers for hyalinization could serve as a validation tool for better understanding the biological behavior of SMAs, and this might lead to better management and treatment. The execution of genetic studies for the prediction of the recurrence in SMAs would also be an interesting prospect to investigate.

## Figures and Tables

**Table 1:** Descriptive features of participants with solid multicystic ameloblastoma.

Characteristics	Frequency	Percentage (%)
Age Groups		
0–20	6	10.0
21–40	49	81.7
41–60	4	6.7
>61	1	1.7
Gender		
Male	34	56.7
Female	26	43.3
Size		
1–2 cm	27	45.0
3–4 cm	9	15.0
>4 cm	24	40.0
Site		
Body of Mandible	38	63.3
Angle of Mandible	9	15.0
Symphysis	2	3.3
Retromolar Trigone	3	5.0
Maxillary Alveolus	8	13.3
Jaw		
Maxilla	8	13.3
Mandible	52	86.7

**Table 2:** Comparison of correlation of SOH with Recurrence of Solid Multicystic Ameloblastoma.

Groups	SOH	Recurrence		χ^2^	*p*-Value
		Absent	Present		
All	Mild	100.0%	0.0%	36.324	0.001 *
	Moderate	100.0%	0.0%		
	Intense	25.0%	75.0%		
Follicular Ameloblastoma	Mild	100.0%	0.0%	11.442	0.003 *
	Moderate	100.0%	0.0%		
	Intense	34.6%	65.4%		
Plexiform Ameloblastoma	Mild	100.0%	0.0%	29.000	0.001 *
	Moderate	100.0%	0.0%		
	Intense	0.0%	100.0%		

Chi—squared test, *p*-value < 0.05—statistically significant. * Indicates significance.

**Table 3:** Comparison of correlation of clinical parameters with recurrence of solid multicystic ameloblastoma. * Indicates significance.

Clinical Parameters		Recurrence		χ^2^	*p*-Value
		Absent	Present		
Site	Body of Mandible	46.3%	53.7%	10.309	0.036 *
	Angle of Mandible	60.0%	40.0%		
	Symphysis	50.0%	50.0%		
	Retromolar Trigone	100.0%	0.0%		
	Maxillary Alveolus	21.6%	0.0%		
Size	1–2 cm	55.6%	44.4%	2.225	0.329
	3–4 cm	40.0%	60.0%		
	>4 cm	66.7%	33.3%		
Jaw	Maxilla	100.0%	0.0%	6.672	0.010 *
	Mandible	51.8%	48.2%		
Radiographic Features	Unilocular Radiolucency	100.0%	0.0%	11.905	0.001 *
	Multilocular Radiolucency	47.1%	52.9%		
Cortical Expansion	Absent	100.0%	0.0%	2.297	0.130
	Present	55.7%	44.3%		
Root Resorption	Absent	100.0%	0.0%	9.693	0.002 *
	Present	49.1%	50.9%		

**Table 4:** Logistic regression of predictor (severity of hyalinization) of recurrence.

Predictor (SOH)	β	S.E.	Wald’s χ^2^	df	*p*-Value	Odd’s Ratio
Mild vs. Severe	1.643	0.544	9.129	1	0.003	5.172
Mild vs. Moderate	1.386	0.703	3.888	1	0.049	4.000
Cox and Snell R2	0.117					
Nagelkerke R2	0.162					
−2 Log likelihood	97.052					

## Data Availability

Not applicable.

## References

[1] Saluja T., Iyer J. (2017). Unmasking the Grey Zone of Hyalinization with a Proposed Classification of Oral Hyalinizing Lesions. J. Interdiscip. Histopathol..

[2] Mukherjee D., Das C., Chatterjee P. (2017). Odontogenic Tumours of Jaw: A Prospective Study on Clinico-Pathological Profile and Their Management. Indian J. Otolaryngol. Head Neck Surg..

[3] Bell R.B., Andersson L., Kahnberg K.E., Pogrel M.A. (2010). Odontogenic and non-odontogenic tumors of the jaws. Oral and Maxillofacial Surgery.

[4] Masthan K.M., Anitha N., Krupaa J., Manikkam S. (2015). Ameloblastoma. J. Pharm. Bioallied. Sci..

[5] Cottom H.E., Bshena F.I., Speight P.M., Craig G.T., Jones A.V. (2012). Histopathological features that predict the recurrence of odontogenic keratocysts. J. Oral Pathol. Med..

[6] Philipsen H.P., Reichart P.A. (2010). Pulse or hyaline ring granuloma. Review of the literature on etiopathogenesis of oral and extraoral lesions. Clin. Oral Investig..

[7] Antony J., Gopalan V., Smith R.A., Lam A.K. (2012). Carcinoma ex pleomorphic adenoma: A comprehensive review of clinical, pathological and molecular data. Head Neck Pathol..

[8] Sathi G.S., Fujii M., Tamamura R., Borkosky S.S., Katase N., Kawakami T., Nagatsuka H., Nagai N. (2008). Juxta-epithelial hyalinization inhibits tumor growth and invasion in ameloblastoma. J. Hard Tissue Biol..

[9] Alturkistani H.A., Tashkandi F.M., Mohammedsaleh Z.M. (2015). Histological Stains: A Literature Review and Case Study. Glob. J. Health Sci..

[10] Wright J.M., Tekkesin M.S. (2017). Odontogenic tumors: Where are we in 2017?. J. Istanb. Univ. Fac. Dent..

[11] Cadavid A.M., Araujo J.P., Coutinho-Camillo C.M., Bologna S., Junior C.A., Lourenço S.V. (2019). Ameloblastomas: Current aspects of the new WHO classification in an analysis of 136 cases. Surg. Exp. Pathol..

[12] Dias C.D., Brandão T.B., Soares F.A., Lourenço S.V. (2013). Ameloblastomas: Clinical-histopathological evaluation of 85 cases with emphasis on squamous metaplasia and keratinization aspects. Acta Odontol. Scand..

[13] Hertog D., Bloemena E., Aartman I.H., van-der-Waal I. (2012). Histopathology of ameloblastoma of the jaws; some critical observations based on a 40 years single institution experience. Med. Oral Patologia Oral y Cirugia Bucal.

[14] Crivelini M.M., Felipini R.C., Miyahara G.I., de Sousa S.C. (2012). Expression of odontogenic ameloblast-associated protein, amelotin, ameloblastin, and amelogenin in odontogenic tumors: Immunohistochemical analysis and pathogenetic considerations. J. Oral Pathol. Med..

[15] Morgan P.R. (2011). Odontogenic tumors: A review. Periodontol. 2000.

[16] Lee S.K., Kim Y.S. (2014). Current Concepts and Occurrence of Epithelial Odontogenic Tumors: II. Calcifying Epithelial Odontogenic Tumor Versus Ghost Cell Odontogenic Tumors Derived from Calcifying Odontogenic Cyst. Korean J. Pathol..

[17] Nicolas J., Magli S., Rabbachin L., Sampaolesi S., Nicotra F., Russo L. (2020). 3D extracellular matrix mimics: Fundamental concepts and role of materials chemistry to influence stem cell fate. Biomacromolecules.

[18] Hinz B., Gabbiani G. (2003). Mechanisms of force generation and transmission by myofibroblast. Curr. Opin. Biotechnol..

[19] Vered M., Shohat I., Buchner A., Dayan D. (2005). Myofibroblasts in stroma of odontogenic cysts and tumors can contribute to variations in the biological behaviour of lesions. Oral Oncol..

[20] Carvalho K.M., Dhupar A., Spadigam A., Syed S. (2017). Ameloblastoma: A 16-year clinicopathological study on Goan population. Indian J. Pathol. Microbiol..

[21] Olaitan A.A., Adeola D.S., Adekeye E.O. (1993). Ameloblastoma: Clinical features and management of 315 cases from Kaduna, Nigeria. J. Craniomaxillofac. Surg..

[22] Au S.W., Li K.Y., Choi W.S., Su Y.X. (2019). Risk factors for recurrence of ameloblastoma: A long-term follow-up retrospective study. Int. J. Oral Maxillofac. Surg..

[23] Kim S.W., Jee Y.J., Lee D.W., Kim H.K. (2018). Conservative surgical treatment for ameloblastoma: A report of three cases. J. Korean Assoc. Oral Maxillofac. Surg..

[24] Sarlabous M., Psutka D.J. (2018). Treatment of mandibular ameloblastoma involving the mandibular condyle: Resection and concomitant reconstruction with a custom hybrid total joint prosthesis and iliac bone graft. J. Craniofacial Surg..

